# Anti-PLA2R antibody measured by ELISA predicts the risk of vein thrombosis in patients with primary membranous nephropathy

**DOI:** 10.1080/0886022X.2022.2057861

**Published:** 2022-04-05

**Authors:** Huizi Zhu, Liang Xu, Xiang Liu, Bing Liu, Chunjuan Zhai, Rong Wang, Xiaowei Yang

**Affiliations:** a Department of Nephrology, Shandong Provincial Hospital, Shandong University, Jinan, China; b Department of Nephrology, Shandong Provincial Hospital Affiliated to Shandong First Medical University, Jinan, China; c Department of Cardiology, Shandong Provincial Hospital affiliated to Shandong First Medical University, Jinan, China

**Keywords:** Primary membranous nephropathy, anti-PLA2R antibody, deep vein thrombosis, risk factors

## Abstract

**Background:**

Primary membranous nephropathy (PMN) is associated with the highest risk for developing venous thrombosis compared with other nephrotic diseases. The aim of the study was to assess the predictive value of the pathognomonic anti-phospholipase A2 receptor (PLA2R) antibody with regard to incidence of venous thrombosis in PMN.

**Methods:**

A total of 365 in-hospital patients diagnosed with PMN were enrolled in the study. Anti-PLA2R antibody was detected by commercial enzyme-linked immunosorbent assay. Multivariate logistic regression was used to detect the independent risk factors for venous thrombosis.

**Results:**

Thirty-seven patients (10.14%) had venous thrombosis. Patients with venous thrombosis had higher levels of cholesterol (CHOL), low-density lipoprotein (LDL), and D-dimer than those without venous thrombosis (*p* < .05). Patients with venous thrombosis had significantly lower levels of albumin (23.95 ± 5.53 vs. 26.18 ± 6.59 g/L, *p* = .049). No significant differences were found in proteinuria, serum creatinine, estimated glomerular filtration rate, platelets, and fibrinogen between patients with and without thrombosis. Anti-PLA2R antibody levels in patients with venous thrombosis were significantly higher than in patients without it (*p* = .002). In the univariate logistic regression, Ln anti-PLA2R antibody (OR: 1.340; *p* = .004), albumin (OR: 0.945; *p* = .050), CHOL (OR: 1.191; *p* = .006), and LDL (OR: 1.271, *p* = .006) were associated with venous thrombosis. Ln anti-PLA2R antibody (OR = 1.269; 95%CI: 1.032–1.561), and LDL (OR = 1.213; 95%CI: 1.017–1.448) were the independent risk factors for venous thrombosis (*p* < .05) in multivariate analysis.

**Conclusions:**

Anti-PLA2R antibody was the independent risk factor for venous thrombosis in PMN. Larger prospective studies were warranted to verify the results in future.

## Background

Deep vein thrombosis (DVT), renal vein thrombosis (RVT), and pulmonary embolism (PE) are collectively known as venous thromboembolism (VTE), which is a common complication and a major cause of morbidity and mortality in patients with nephrotic syndrome (NS) [1,2].

The underlying pathophysiological mechanisms of VTE in patients with NS have not been fully elucidated. Hypercoagulability is postulated to due to a variety of elements, including the imbalance of glomerular loss of coagulation factors with increased liver procoagulants synthesis, altered platelet activity, intravascular volume contraction, venous stasis accompanying edema [3,4]. Proteinuria and serum albumin levels, which reflect the severity of NS, are the most frequently studied VTE risk factors in patients with NS [5–7]. Higher proteinuria and lower serum albumin levels have been suggested to be risk factors of VTE in some but not all studies [7–10]. Compared with other nephrotic diseases, membranous nephropathy (MN) is associated with the highest risk for developing venous thrombosis, especially RVT [11,12], even adjusted for gender, proteinuria, and serum albumin by multivariable analysis [13]. The reason for the increased thromboembolic risk in MN has yet to be unraveled.

Identification of antibody to phospholipase A2 receptor (PLA2R) in 70–80% of adult patients with primary MN (PMN) is the major breakthrough, which shows PMN is an autoimmune disease [14,15]. Anti-PLA2R antibody is highly specific for MN, rarely being detected in other nephropathies, autoimmune diseases, or healthy individuals. Furthermore, a number of studies have shown that PLA2R autoantibody correlate closely with disease activity and progression [16,17] and can be used to monitor response to immunosuppressive therapy [18,19]. Since anti-PLA2R antibody was considered to be pathogenic for MN, some researchers have postulated the association between anti-PLA2R antibody and the particularly high VTE risk [13]. Moreover, in our clinical experience, we observed some patients with serum albumin level around 30 g/L and extremely high level of anti-PLA2R antibody had extensive VTE. To the best of our knowledge, there was no clinical study to evaluate whether anti-PLA2R antibody was the risk factor for VTE in patients with PMN till now.

In this study, we added anti-PLA2R antibody to the common thrombophilic risk factors, such as proteinuria, hypoalbuminemia, and serum creatinine, to analyze the predictors of venous thrombosis in a large Chinese PMN cohort.

## Methods

### Design

We retrospectively reviewed all the in-hospital patients diagnosed with PMN in Shandong Provincial Hospital affiliated to Shandong First Medical University between January 2018 and January 2021. The prevalence of venous thrombosis and its risk factors was investigated at the time of diagnosis. Follow-up data were collected by review of the hospital's electronic medical records.

### Participants

The inclusion criteria for PMN were as follows: biopsy-diagnosed MN or patients with positive anti-PLA2R antibody test, except for clinical factors such as systemic lupus erythematosus (SLE), hepatitis B virus (HBV), tumor, and drug-induced secondary MN [20]. Major exclusion criteria included: (1) without the result of anti-PLA2R antibody test; (2) without a venous examination; (3) use of immunosuppressive drugs prior to the study enrollment; (4) exposure to classic risk factors for venous thrombosis such as antiphospholipid syndrome, major surgery, prolonged immobilization.

The Ethics Committee of Shandong Provincial Hospital affiliated to Shandong First Medical University approved the study.

### Venous thrombosis examination

All the patients enrolled in the study received renal vascular and lower extremity vascular ultrasounds for venous thrombosis screening. Venous ultrasound was screened by sonologists with 10-year experience in vessel ultrasound. Two patients received computed tomography pulmonary angiography (CTPA) and diagnosed with PE. Both of them had extensive renal venous and inferior vena cava thrombosis.

### Clinical evaluation

Clinical data were obtained at the time of venous examination by reviewing the patient's previous medical records, including age, sex, blood pressure measurements, history of smoking, hemoglobin, platelet count, serum albumin, serum lipids profile, plasma fibrinogen, D-dimer, urinalysis, quantification of proteinuria, blood urea nitrogen, serum creatinine, estimated glomerular filtration rate (eGFR), anti-PLA2R antibody, and pathological results. In our cohort, anti-PLA2R antibody was detected by commercial enzyme-linked immunosorbent assay (ELISA), and the manufacturer’s recommended cutoff value is 20 RU/mL.

Complete remission (CR) was defined as ≤0.3 g/day proteinuria plus stable renal function, partial remission (PR) as a 50% reduction of initial proteinuria, and less than 3.5 g/day with stable renal function.

### Statistical analysis

Statistical software SPSS 25.0 (IBM SPSS Statistics for Windows, IBM Corp., Armonk, NY) was employed for statistical analysis. Data with a normal distribution were expressed as mean ± standard deviation (SD) and compared by *t*-tests, and data with a non-normal distribution were presented as the median and quartile and compared by nonparametric test. The categorical variables were expressed as rates and were compared by the *χ*^2^ test. The correlation between two parameters (nonparametric distributions) was analyzed by Spearman’s rank coefficient of correlation. Univariate analysis followed by multivariate logistic regression analysis with stepwise variable selection procedure was applied to identify independent factors associated with vein thrombosis event. All baseline variables entered the initial model and were maintained if *p* < .05. Statistical significance was considered as *p* < .05.

## Results

### Patient population

A total of 365 in-hospital patients diagnosed with PMN at Shandong Provincial Hospital affiliated to Shandong First Medical University from January 2018 to August 2021 were enrolled in the study, including 355 biopsy-proven PMN and 10 anti-PLA2R antibody positive patients who did not receive renal biopsy because of extensive venous thrombosis. All of the 10 patients without renal biopsy received associated evaluation to exclude clinical conditions such as SLE, HBV, tumor, and drug-induced secondary MN. Of the 10 patients, the average albumin levels were 22.0 ± 4.4 g/L, the average 24 h urine protein were 7.56 ± 4.14 g, and the anti-PLA2R antibody levels ranged from 77.22 to 1500 RU/mL, with a median value of 173.89 RU/mL at time of diagnosis.

### General baseline characteristics of the study patients

General baseline clinical profiles of the patients are listed in Table 1. Of the 365 patients, 135 (36.99%) were females and 230 (63.01%) were males. The mean age of the patients was 47.69 ± 12.01 years. 67.67% (247/365) of the patients had nephrotic-level proteinuria. At a cut-off value of 20 RU/mL for anti-PLA2R antibody, 83.33% (293/365) of the patients had either elevated anti-PLA2R antibody in the serum or enhanced PLA2R in glomeruli. In Spearman’s correlation analysis, anti-PLA2R antibody correlated positively with proteinuria (*r* = 0.296, *p* < .001), total cholesterol (CHOL) (*r* = 0.255, *p* < .001), and serum creatinine (*r* = 0.112, *p* = .033). The anti-PLA2R antibody level correlated negatively with serum albumin (*r* = −0.337, *p* < .001).

**Table 1.** The clinical parameters of the studied PMN patients.

Parameters	*n* = 365
Gender (male/female)	230/135
Age (mean ± SD) (years)	47.90 ± 12.01
Level of PLA2R Abs (median, quartile) (RU/mL)	37.55 (4.17, 136.40)
<2 (*n*, %)	52 (14.25)
2–20 (*n*, %)	102 (27.95)
>20 (*n*, %)	211 (57.81)
Ln PLA2R Abs (mean ± SD)	3.36 ± 1.87
Glomerular PLA2R antigen positive (*n*, %)	265 (72.59)
PLA2R-related MN^a^ (*n*, %)	293 (83.33)
Urinary protein (mean ± SD) (g/24 h)	6.43 ± 4.82
Nephrotic proteinuria (*n*, %)	247 (67.67)
Albumin (mean ± SD) (g/L)	25.95 ± 6.52
SCR (mean ± SD) (μmol/L)	67.75 ± 30.53
eGFR (mean ± SD) (mL/min/1.73 m^2^)	107.37 ± 20.69
CHOL (mean ± SD) (mmol/L)	7.98 ± 2.46
LDL (mean ± SD) (mmol/L)	5.16 ± 1.83
Combined with DM (*n*, %)	39 (10.68)

PMN: primary membranous nephropathy; SD: standard deviation; PLA2R: phospholipase A2 receptor; Abs: antibodies; SCR: serum creatinine; eGFR: estimated glomerular filtration rate; CHOL: cholesterol; LDL: low-density lipoprotein; DM: diabetes.

^a^ PLA2R-related MN was defined as either positive for serum anti-PLA2R antibody (cutoff value of 20 RU/mL) or glomerular PLA2R antigen.

**Figure 1.**
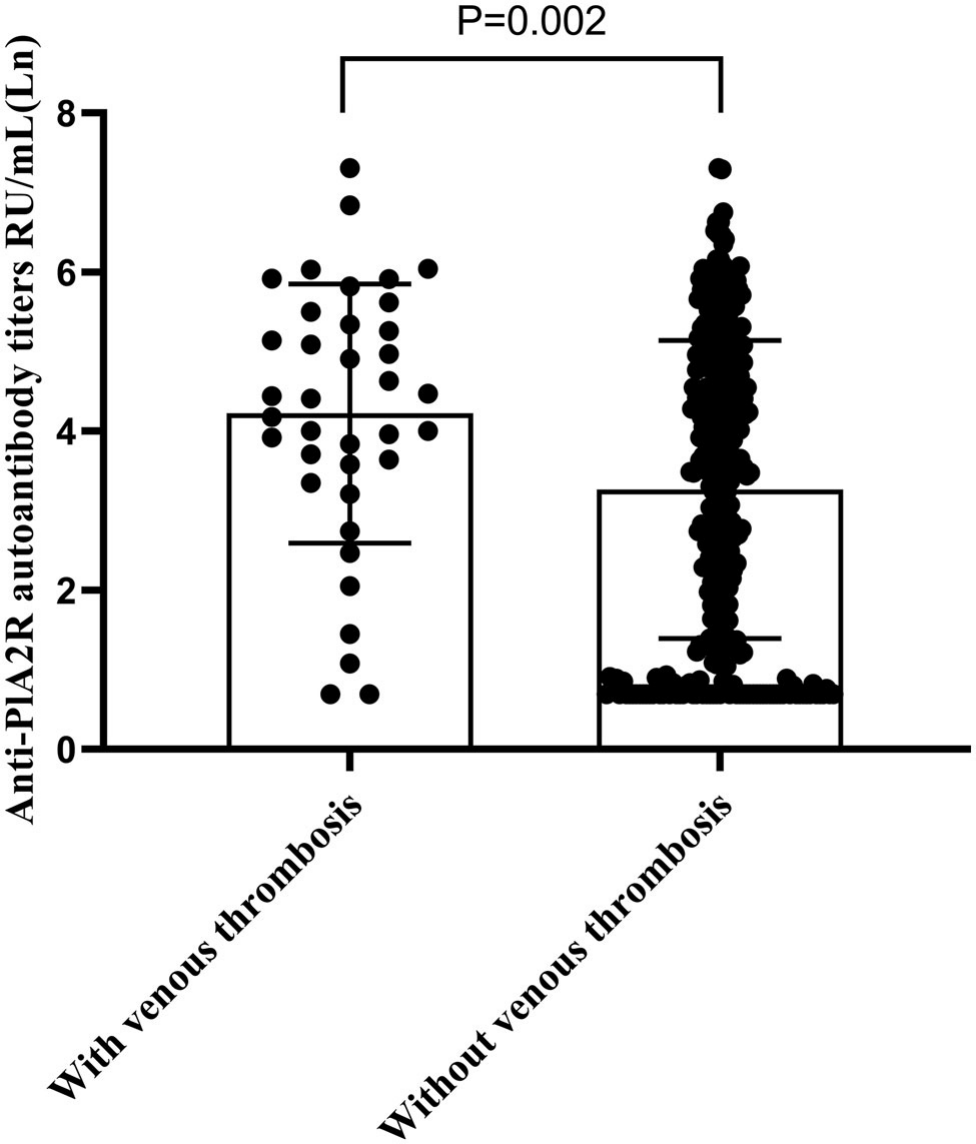
Scatter plot shows the levels of anti-PLA2R antibody in PMN patients with and without venous thrombosis. Anti-PLA2R antibody was highly skewed, so natural log transformation was used for the analysis.

Among the studied patients, 37 patients (10.14%) had venous thrombosis. A total of 46 anatomic site venous thrombosis were detected in these patients, and seven patients had simultaneous venous thrombosis at more than one site. Twenty-two patients had a DVT; 11 patients had a RVT; 10 patients had an inferior vena cava thrombosis; two patients had a PE. The clinical characteristics of patients with and without venous thrombosis at the time of vascular ultrasounds examination are shown in Table 2. There were no statistical differences in the distributions of age and sex between patients with and without venous thrombosis. Patients with venous thrombosis had significantly higher level of CHOL, low-density lipoprotein (LDL), D-dimer, and lower level of albumin than those without venous thrombosis (*p* = .025, *p* = .037, *p* < .001, *p* = .049, respectively). No significant differences were found in urine proteinuria, SCR, eGFR, platelets, and fibrinogen between patients with and without thrombosis. Anti-PLA2R antibody was highly skewed, in Mann–Whitney’s *U*-test anti-PLA2R antibody level in patients with venous thrombosis were significantly higher than in patients without it (*p* = .004). The distributions of Ln anti-PLA2R antibody of the two groups are shown in Figure 1.

**Table 2.** Laboratory findings of PMN with or without venous thrombosis.

Parameters	With venous thrombosis (*n* = 37)	Without venous thrombosis (*n* = 328)	*p* Value
Age (mean ± SD) (years)	48.97 ± 10.94	47.78 ± 12.14	.567
Gender (female, %)	10 (27.03%)	125 (38.11%)	.186
PLT (mean ± SD) (×10^9^/L)	274.47 ± 67.43	266.31 ± 62.51	.462
Ln PLA2R Abs (mean ± SD)	4.22 ± 1.63	3.26 ± 1.87	**.002**
Urinary protein (mean ± SD) (g/24 h)	7.20 ± 4.25	6.34 ± 4.87	.320
Albumin (mean ± SD) (g/L)	23.95 ± 5.53	26.18 ± 6.59	**.049**
SCR (mean ± SD) (μmol/L)	71.19 ± 18.97	67.36 ± 31.56	.470
eGFR (mean ± SD) (mL/min/1.73 m^2^)	104.03 ± 18.15	107.75 ± 20.95	.301
CHOL (mean ± SD) (mmol/L)	9.07 ± 3.00	7.86 ± 2.37	**.025**
LDL (mean ± SD) (mmol/L)	5.98 ± 2.46	5.07 ± 1.72	**.037**
D-dimer (median, quartile) (μg/mL)	1.41 (0.57, 3.41)	0.57 (0.34, 1.06)	**<.001**
Fib (median, quartile) (g/L)	4.09 (3.53, 4.86)	3.97 (3.50, 4.67)	.518
Combined with DM (*n*, %)	3 (8.11%)	36 (10.98%)	.592

PMN: primary membranous nephropathy; SD: standard deviation; PLT: platelet; PLA2R: phospholipase A2 receptor; Abs: antibodies; SCR: serum creatinine; eGFR: estimated glomerular filtration rate; CHOL: cholesterol; LDL: low-density lipoprotein; Fib: fibrogen; DM: diabetes.

Bold values are statistical significance.

### Risk factors of venous thrombosis in patients with PMN at time of diagnosis

**Figure 2.**
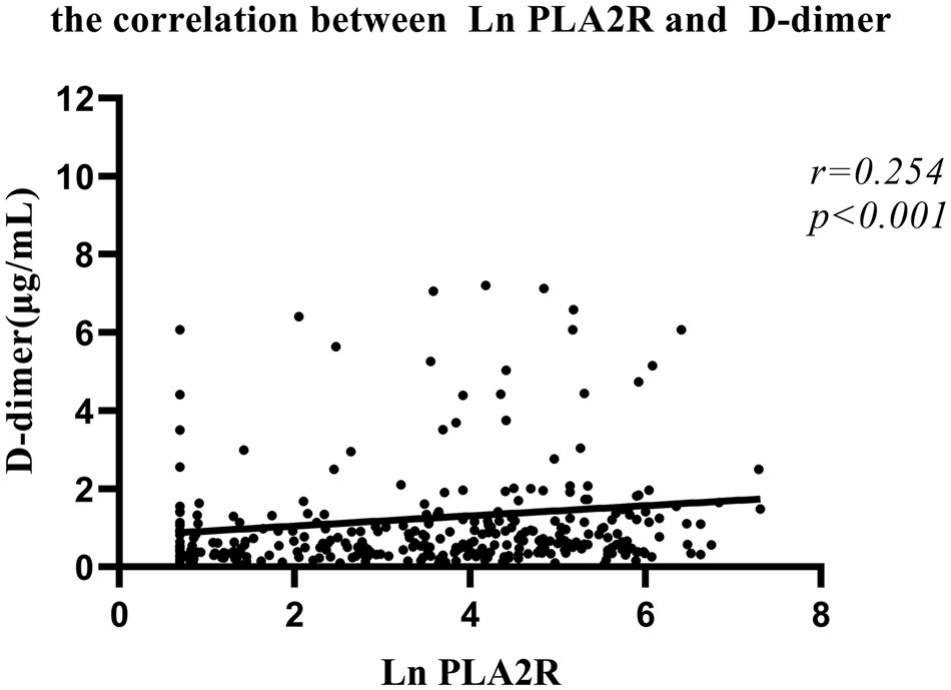
Scatter plot shows the correlation between the levels of anti-PLA2R antibody and the levels of plasma D-dimer in Spearman’s correlation analysis. Anti-PLA2R antibody was highly skewed, so natural log transformation was used for the analysis.

D-dimer is a fragment of degraded fibrin reflecting the activation of fibrinolysis and thrombosis, which was used as the marker of hypercoagulable state in patients with MN [21]. In our patients, Spearman’s correlation analysis showed that higher anti-PLA2R antibody level was positively and significantly correlated with the plasma D-dimer level (Figure 2; *r* = 0.254, *p* < .001).

Since plasma D-dimer level was influenced by many factors, correlation of anti-PLA2R antibody and venous thrombosis was further analyzed. Results of the univariate and multivariate logistic regression analyzing risk factors for venous thrombosis in PMN are shown in Table 3. Anti-PLA2R antibody was highly skewed, so natural log transformation was used for the analysis. In the univariate logistic regression, Ln anti-PLA2R antibody (OR: 1.340; *p* = .004), albumin (OR: 0.945; *p* = .050), CHOL (OR: 1.191, *p* = .006), and LDL (OR: 1.271, *p* = .006) were associated with venous thrombosis. CHOL was highly colinear with LDL, and then albumin, LDL, and Ln anti-PLA2R antibody were used in the further multivariate logistic regression with forward-conditional method. We found that only Ln anti-PLA2R antibody (OR = 1.269; 95%CI: 1.032–1.561), and LDL (OR = 1.213; 95%CI: 1.017–1.448) were the independent risk factors for venous thrombosis (*p* < .05).

**Table 3.** Risk factors in predicting venous thrombosis in patients with PMN.

Parameters	Univariate analysis		Multivariate analysis	
	*p* Value^a^	OR (95%CI)	*p* Value	OR (95%CI)
Age per year	.566	1.008 (0.980, 1.038)		
Sex (female)	.189	0.601 (0.282, 1.285)		
Ln PLA2R Abs per Ln change	**.004**	**1.340 (1.098, 1.636)**	**.024**	**1.269 (1.032, 1.561)**
Albumin per g/L	**.050**	**0.945 (0.892, 1.000)**		
SCR per μmol/L	.477	1.003 (0.994, 1.012)		
eGFR per mL/min/1.73 m^2^	.301	0.992 (0.977, 1.007)		
PLT per ×10^9^/L	.461	1.002 (0.997, 1.007)		
CHOL per mmol/L	**.006**	**1.191 (1.050, 1.351)**		
LDL per mmol/L	**.006**	**1.271 (1.073, 1.506)**	**.032**	**1.213 (1.017, 1.448)**
Proteinuria per g/day	.321	1.033 (0.968, 1.103)		
Fib per g/L	.730	1.028 (0.877, 1.206)		
Combined with DM	.594	0.716 (0.209, 2.449)		

PMN: primary membranous nephropathy; PLA2R: phospholipase A2 receptor; Abs: antibodies; SCR: serum creatinine; eGFR: estimated glomerular filtration rate; PLT: platelet; CHOL: cholesterol; DM: diabetes; Fib: fibrogen.

^a^ Only variables with *p*≤ .05 in the univariate logistic regression analysis were used in the multiple logistic regression.

^a^ Bold values are statistical significance.

### Follow up

Follow-up data were collected by review of the hospital's electronic medical records. Two hundred and thirty-five of the 365 patients were followed up in our in-patient or out-patient clinics for at least 3 months, with a median follow-up time of 11 months (3–42 months).

Among the 235 patients, 29 patients were diagnosed with venous thrombosis at baseline (two with PE, 13 with RVT and/or inferior vena cava thrombosis, 14 with DVT), with a median follow-up time of 16 months (3–39 months). All of the 29 patients received immunosuppressive therapy, and low-molecular-weight heparin (LMWH) for 2–4 weeks followed by oral warfarin or rivaroxaban planning for at least six months. During the follow-up, 25 patients reached CR or PR with a significant decline of the level of anti-PLA2R antibody (269.44 ± 380.22 vs. 10.8 ± 19.7 RU/mL; *p* < .01). Among the 25 patients, thrombosis was absent in six patients, partially resolved in eight patients, 11 did not receive a second ultrasound examination. Four of the 29 patients experienced no response in proteinuria, but the thrombosis was partially resolved with persistently anti-coagulation therapy. Among the four patients, anti-PLA2R antibody of one patient turned negative, while anti-PLA2R antibody of the other three patients maintained positive.

One hundred and ninety-six patients without thrombosis at baseline had follow-up data. One hundred and forty-one patients received immunosuppressive therapy, while the others received supportive care only at baseline. According to local clinical practice, patients with albumin lower than 30 g/L received anti-coagulation therapy, such as aspirin, LMWH, warfarin, and rivaroxaban. During the follow-up, 131 patients reached CR or PR. Thirteen patients received a second venous thrombosis examination, and one patient presented with new thromboembolic events, who experienced no response in proteinuria after 4 month immunosuppressive treatment with anti-PLA2R antibody level increasing from 288.12 to 345.14 RU/mL.

## Discussion

Compared with other nephrotic diseases, PMN is associated with the highest risk for developing venous thrombosis [11–13]. The aim of this study was to explore whether the pathognomonic anti-PLA2R antibody contributes to venous thrombosis risk in PMN.

In this large PMN cohort, venous thrombosis occurred in 10.14% of the patients, and DVT occurred more frequently than RVT. The frequency of venous thrombosis in our patients was consistent with some reports [13,22], but substantially lower than studies using more sensitive examination for VTEs screening, such as CTPA or lung ventilation and perfusion scintigraphy [10,23]. In our local clinical practice, considering the risk of contrast induced nephropathy, CTPA was not performed unless patients presented with clinical signs and symptoms of PE, such as dyspnea, hemoptysis, chest pain, and syncope. Lung ventilation and perfusion scintigraphy was neither routinely performed. Hence, in this study, we focused on venous thrombosis and symptomatic PE. The other explanation for the relatively lower prevalence of venous thrombosis was that non-nephrotic patients were also included and accounted for 32.33% in our cohort. Although the prevalence of thrombosis was lower in patients without NS than in patients with it, two venous thrombosis events were indeed detected in patients without NS. Non-nephrotic patients should also be involved to explore the risk predictors of thrombosis in PMN.

In our cohort, we found that hypoalbuminemia, CHOL, and LDL were the risk factors for venous thrombosis in univariate analysis. Proteinuria was not the predictive of thrombotic events in this cohort. Anti-PLA2R antibody is an organ-specific autoantibody which targets the kidney podocytes, and is considered to be pathogenic in PMN. The levels of anti-PLA2R antibody correlate closely with disease activity and progression in PMN [16,17]. In the cross-sectional analysis, anti-PLA2R antibody was correlated with proteinuria and hypoalbuminemia at baseline. More interestingly, the levels of anti-PLA2R antibody were significantly higher in patients with venous thrombosis than that of patients without venous thrombosis. Furthermore, in multivariate logistic regression analysis, anti-PLA2R antibody was the independent risk factor for venous thrombosis, even adjusted for albumin and LDL. The results of our study indicated that anti-PLA2R antibody is superior to albumin and proteinuria in relation to the assessment of the risk of venous thrombosis in PMN. In this retrospective study, only 27 patients received a second venous thrombosis examination during follow up. Among the 27 patients, only one new thromboembolic event was detected. In spite of the limited follow-up data, the lower frequency of new thrombosis might be due to extensive anti-coagulation therapy in patients with PMN to some extent. The dynamic effect of anti-PLA2R antibody on venous thrombosis needs to be identified in larger long-term prospective studies.

The high risk of VTE in individuals with nephrotic range proteinuria was assumed to be secondary to loss of anticoagulant proteins. However, microalbuminuria and declined eGFR were also found to be independently associated with increased risk for VTE [24–27]. Pang et al. performed urine proteomics of PMN and found that these proteins are mainly involved in immune response and coagulation cascades [28]. These results indicated that there might be direct links between renal injury and thrombosis. The correlation of anti-PLA2R antibody induced renal injury and activation of coagulation deserves further investigation. On the other hand, a recent study demonstrated that MN patients with Th17-mediated inflammation had more VTEs [29]. Th17-immune response is a proinflammatory immune pathway associated with autoimmune diseases [30,31]. Whether the development of thrombosis will be induced by the activation of autoimmune response against PLA2R is another question for future consideration.

This study has several limitations. First, to explore the role of anti-PLA2R antibody in the thrombosis risk, it would be the best to investigate it in PLA2R-related MN. The optimum cut-off value for the diagnostic accuracy of the commercially available ELISA assay in different populations was still under discussion [32,33]. Moreover, in patients who are anti-PLA2R antibody negative, the positive staining of the renal biopsy for PLA2R antigen may also disclose PLA2R-related MN [34]. It is hard to accurately define the PLA2R-related MN. However, in our cohort, using a relatively conservative cut-off value of 20 RU/mL for anti-PLA2R antibody, 83.33% of the patients had either elevated anti-PLA2R antibody in the serum or enhanced PLA2R in glomeruli. That is, PLA2R-related MN accounted for the majority of the population in our cohort. Second, despite the large size of our cohort, there were relatively few events, which might limit the ability to identify predisposing risk factors. Finally, based on a retrospective analysis of passively captured clinical events, the true frequency might be underestimated and the dynamic effect of anti-PLA2R antibody on venous thrombosis cannot be determined.

## Conclusions

In summary, we were the first to explore the risk of anti-PLA2R antibody in the development of venous thrombosis in PMN, and found anti-PLA2R antibody to be the independent risk factor. Larger prospective studies are warranted to verify the results in future.

## Data Availability

The datasets used during the current study are available from the corresponding author on reasonable request.

## Abbreviations

CHOL: cholesterol
CTPA: computed tomography pulmonary angiography
CR: Complete remission
DVT: deep vein thrombosis
eGFR: estimated glomerular filtration rate
ELISA: enzyme-linked immunosorbent assay
HBV: hepatitis B virus
LDL: low-density lipoprotein
MN: membranous nephropathy
NS: nephrotic syndrome
PE: pulmonary embolism
PLA2R: phospholipase A2 receptor
PMN: primary membranous nephropathy
SLE: systemic lupus erythematosus
RVT: renal vein thrombosis
VTE: venous thromboembolism
PR: partial remission
SD: standard deviation
